# Simulation of the Effects of Forest Management and Water Protection Practices on Nutrient Exports in a Forested Boreal Catchment

**DOI:** 10.1007/s00267-025-02273-4

**Published:** 2025-09-23

**Authors:** Mika Nieminen, Aleksi Räsänen, Janne Miettinen, Sakari Sarkkola, Leena Stenberg, Timo Pukkala

**Affiliations:** 1 Natural Resources Institute Finland, Helsinki, Finland; 2 Natural Resources Institute Finland, Oulu, Finland; 3 Faculty of Science and Forestry, School of Forest Sciences, University of Eastern Finland, Joensuu, Finland

**Keywords:** Continuous cover forestry, Rotation forestry, Nutrients, Water quality, Wetland buffer

## Abstract

There is an urgent need to improve water quality management in forested catchments, particularly in forestry-drained peatland areas. We utilized nutrient export models and forestry simulations to forecast the impact of forest management and water protection practices on nitrogen and phosphorus exports from forests to waters in the Kiiminkijoki catchment area, central Finland. Our simulations indicated that the choice between forest management systems (even-aged forestry, extended rotation length, continuous cover forestry, no forestry) may have a larger impact on nutrient exports from mineral soil forests than from drained peatland forests. Of the water protection practices, sedimentation ponds, peak runoff control dams, and riparian buffer zones may have little effect on nutrient exports, but wetland buffers in drained peatland forests may reduce nutrient exports to a significantly lower level. Our simulations suggested that forestry operators should consider continuous cover forestry and wetland buffers when trying to improve water quality in forested catchments.

## Introduction

When compared to point-source pollution and agricultural diffuse pollution, forestry has previously been considered a relatively small source of nutrients to surface waters (Finér et al. 2010). However, recent studies suggest that forestry, particularly on peatlands, results in significantly more profound and longer-lasting nutrient exports than previously thought (Nieminen et al. 2017b, 2018c, 2021; Finér et al. 2021). In Finland, for example, earlier studies have estimated that forestry on peatlands accounts for only 1–3 percent of all human-induced nutrient exports (Finér et al. 2010), but recent studies suggest that this percentage may be as much as 15–20% (Nieminen et al. 2020a).

Many studies have further indicated increasing temporal trends in dissolved carbon and nutrient concentrations in waters discharging from drained peatland forests (Nieminen et al. 2017b, 2018c, 2021; Asmala et al. 2019; Räike et al. 2019). To meet the targets of national and international water quality protection programs, such as the EU Water Framework Directive, there is an urgent need to improve water quality management in forested catchments. This is particularly true for regions where forestry-drained peatlands constitute a large proportion of the landscapes, such as Finland, Sweden, Estonia, and large parts of the British Isles.

Water quality protection in forested areas has traditionally focused on water protection structures that aim to capture nutrients and sediments before they enter the receiving water body. However, many of these structures are inefficient in reducing sediment and nutrient exports (Haahti et al. 2018). Some of the structures, such as sedimentation ponds and pits, often increase rather than decrease sediment exports due to erosion of the soils exposed during their construction (Joensuu et al. 1999; Haahti et al. 2018). Moreover, most of the water protection structures only retain sediments and particulate nutrients and do not affect dissolved nutrients (Nieminen et al. 2018b). Therefore, they may be effective in mitigating the water quality impacts of drainage and mechanical site preparation operations, but they do not decrease nutrient loads from forest fertilization and harvesting.

The only widely implemented water protection practices that have any effect on dissolved nutrients are traditional buffer zones alongside streams and lakes, and natural and restored wetland buffers. However, while the importance of buffer zones for the biodiversity of streams and their riparian areas is unquestionable (Mykrä et al. 2023), their role in decreasing nutrient exports may not be particularly significant. The study by Miettinen et al. (2020) indicated that traditional 5–20 m wide buffer zones only retain 10–20% of the nutrients discharging from forest clear-cut sites. On the other hand, wetland buffers may be highly efficient as they may retain almost all nutrients from their throughflow waters (Väänänen et al. 2008; Vikman et al. 2010). A significant problem related to wetland buffers is, however, that there are seldom suitable natural wetlands to be used as buffer areas, as such sites have often been drained for forestry, agriculture, or peat mining purposes (Fluet-Chouinard et al. 2023). If a drained site is restored to be used as a wetland buffer by filling in or blocking its ditches, a problem is that restoration initially increases rather than decreases nutrient exports (Sallantaus et al. 1998; Nieminen et al. 2020b).

The problems related to different water quality protection practices have raised the question, whether it would be more reasonable to decrease forestry-derived nutrient exports than to focus on capturing the nutrients released to surface waters by forest management. An efficient means to decrease forestry-derived nutrient exports could be continuous cover forestry (CCF), instead of the prevailing rotation forestry (RF) management (Nieminen et al. 2018a; Härkönen et al. 2023). CCF is thought to decrease nutrient exports relative to RF management, particularly because it avoids large clear-cuts and eliminates the need for regular ditch cleanings in drained peatland forests (Nieminen et al. 2018a). In addition, lengthening the rotation period in RF could decrease nutrient exports by decreasing the frequency of clear-cuts. However, there have been no studies that compare the efficiency of forest management and water protection practices in nutrient export mitigation.

A challenge in comparing different forest management methods in managing nutrient exports is that they may result in differing harvest volumes. While one method may yield lower nutrient exports per unit area, it may not necessarily represent a more environmentally sustainable option for supplying wood to the forest industry if it requires harvesting over a substantially larger area. Therefore, when evaluating different management strategies, it is essential to consider nutrient export not only on an area basis but also concerning the volume of harvested biomass.

It is not possible to study the effects of multiple water protection practices and silvicultural systems on nutrient exports empirically at the same space and time, as the effects of one practice cannot be separated from those of the other ones. However, empirical information collected in earlier studies can be used to model the effects of water protection practices and silvicultural systems. These models can then be used in simulation studies to predict the consequences of alternative management and water protection options.

The present study simulates the future development of forests in a large boreal catchment under different forest management options and quantifies associated nitrogen and phosphorus exports and the efficiencies of different water protection practices. This is achieved by using empirically driven nutrient export equations and process-based modeling for different forest operations and water protection practices. We first simulate nutrient exports with the current national recommendations for RF management in Finnish forests (Äijälä et al. 2014) and then introduce different management options to decrease those exports. These options involve managements that have received attention as possible solutions to steer the current dominant RF-based management in a more environmentally friendly direction, such as CCF and extending the rotation length. Second, we will simulate how much different water protection practices (sedimentation ponds, peak runoff control dams, riparian buffer zones, wetland buffers) decrease nutrient exports compared with introducing environmentally friendly forest management options. The main aim of our simulation is to indicate which forest management options and water protection practices should be prioritized in operational forestry when improving water quality management in forested catchments.

## Material and Methods

### Site Description

We simulated the future development of forests and associated nutrient exports of different forest operations for the Kiiminkijoki catchment area in central Finland (3824 km^2^, 64° 40’–65° 18’ N, 25° 17’–28° 02’ E). To clarify our calculation procedure, a flow chart of calculations is given in an appendix (Appendix Fig. 6).

Between 1991 and 2020, the average temperature in Kiiminkijoki was 3.3 °C, with means of −8.4 °C in February and 16.7 °C in July. The region receives an average precipitation of approximately 524 mm (Oulu airport weather station; Jokinen et al. 2021). The land use in the catchment is dominated by forests on mineral soils (40%), forestry-drained peatlands (31.5%) and undrained open and forested peatlands (21.1%), while smaller proportions belong to water bodies (3.5%), agricultural areas (1.8%), built-up areas (1.5%), and present or former peat harvesting areas (0.6%) (Sarkki et al. 2023). The Kiiminkijoki catchment area forests are mostly in commercial use. The protected areas (11% of land area) are predominantly low-productive pristine mires.

The ecological water quality in the river and its tributaries varies from poor to excellent, with good quality downstream of the river (Finnish Environment Institute 2023). Among local inhabitants and stakeholders, there is a strong desire to improve the water quality, particularly to enable the return of salmon and other migratory fish which have disappeared almost entirely due to the deteriorated water quality and strong discharge variation caused by extensive peatland drainage and other intensive land use in parts of the catchment (Sarkki et al. 2023).

### Estimation of Nutrient Exports

#### Drainage Legacy and Forest Management Operations

We calculated seven scenarios for forest management (four scenarios for mineral soils and three for drained peatlands) and four scenarios for water protection practices (Tables 1 and 3). Extended rotation period (ERP) scenario was not calculated for drained peatland forests, as CCF is considered a more efficient strategy for mitigating nutrient exports and greenhouse gas emissions (Nieminen et al. 2018a; Shanin et al. 2021; Härkönen et al. 2023). Consequently, CCF is regarded the principal management approach for future peatland forests. For further information on the calculation of scenarios, see the section “Simulation of scenarios”.

**Table 1:** Calculated scenarios (indicated by x) for forest management in mineral soil (upland) forests and drained peatland forests in the Kiiminkijoki catchment

Scenario No. for forest management	Forest management	Mineral soil forests	Drained peatland forests
1	Rotation forestry (RF) according to current recommendations (Äijälä et al. 2014)	x	x
2	Extended rotation period (ERP)	x	
3	Continuous cover forestry (CCF)	x	x
4	No forestry operations	x	x

For further information on the calculation of scenarios, see the text.

Calculation of N and P exports from the seven forest management scenarios were based on the perception that forestry increases nutrient exports due to long-term legacy effect of past peatland drainage operations (Nieminen et al. 2017b, 2018c, 2021, Räike et al. 2019), as well as the shorter-term effects of ditch network maintenance (DNM), forest fertilization, and harvesting. The details of the procedure for calculating forestry-induced nutrient exports have been reported previously in Finnish in Nieminen et al. (2023).

The calculation of the long-term legacy effect of drainage assumes that it is controlled by nutrient mineralization (Nieminen et al. 2022), which, in turn, is influenced by site drainage conditions, specifically the soil water table level (WTL) (Ojanen and Minkkinen, 2019). Estimation of the legacy effect of drainage began by predicting the WTL for all forest compartments representing drained peatland forests under late summer (August) conditions. This was done by using the empirical equation by Sarkkola et al. (2010):

1 $${\text{WTL}}=47.92+159.83{(1-0.3}^{\frac{V}{90}})-30.684\left(1-{0.3}^{\frac{V}{90}}\right){\text{ln}}\left(S\right)+8.095{\text{ln}}\left(D\right)-0.73{LAT}-0.185S$$

where WTL is the water level during August (cm), *V* is the growing stock volume (m^3^ ha^−1^), *S* is the mean monthly precipitation (mm) in summer (July-August), *D* is the average ditch depth (cm), and *LAT* is the northern latitude (degrees). The data by Sarkkola et al. (2010) consist of measurements of WTL and tree stand characteristics in 460 sample plots in drained peatland forest stands in Finland. In our simulations, the average ditch depth was assumed to be 60 cm, which is the typical depth for ditches in areas where >10 years have elapsed since the previous DNM operation (Hökkä et al. 2020).

The relationship between WTL and N and P exports due to peat mineralization (i.e., the legacy effect of drainage) was calculated by the SUSI peatland simulator (Laurén et al. 2021). SUSI has been widely used in recent studies to simulate forest growth and biogeochemical processes in drained peatland forests. SUSI describes hydrology, biogeochemical processes and stand growth along a 2D cross-section between two parallel ditches in a drained peatland forest. Nutrient exports in SUSI arise from aerobic peat mineralization, and because the nutrients released upon mineralization from the peat below the rooting zone (here 0.4 m) of trees and other vegetation are not adsorbed by soil or accumulated in vegetation but are exported to ditches. According to SUSI simulations, as also indicated by earlier empirical studies (Nieminen et al. 2022), nutrient exports are particularly high from mature stands (high evapotranspiration capacity) as their low water levels enhance aerobic peat mineralization in deep peat layers. The relationships between WTL and the nutrient exports simulated by SUSI for drained peatlands of differing fertility are shown in Nieminen et al. (2023) and the appendix (Appendix Fig. 7).

The shorter-term effects of DNM, fertilization, and harvesting (clear-cuts) of mineral soil forests on N and P exports were estimated by using export coefficients, which describe how much each forestry operation increases nutrient exports per treatment area and year (Table 2). For fertilization, we used the coefficients by Finér et al. (2010), and for mineral soil forest harvesting, the coefficients by Nieminen et al. (2023). The effects of DNM on N and P exports were calculated based on the findings that DNM does not increase dissolved N and P exports (Joensuu et al. 2002, Nieminen et al. 2010), but increases the exports of N and P adhered to suspended solids. Accordingly, DNM-induced nutrient exports were calculated as the product of suspended solid exports and the concentrations of N and P in suspended solids. Export coefficients for DNM-induced suspended solids exports were derived from Finér et al. (2010) and the concentrations of N and P in suspended solids from Nieminen et al. (2017a). The suspended solid export coefficients for DNM by Finér et al. (2010) were modified by excluding the 30% decrease in exports by water protection practices, that is, their coefficients were multiplied by about 1.43 (Table 2).

**Table 2:** *N* and *P* export coefficients (kg ha^−1^ year^−1^) for fertilization of mineral soil forests and drained peatland forests, as well as for DNM in drained peatland forests and clear-cutting in mineral soil forests

Years from treatment	DNM, drained peatlands^a^		Fertiliz., mineral soils^b^	Fertiliz., drained peatlands^b^	Clear-cut, mineral soil forests^c^	
	*N*	*P*	*N*	*P*	*N*	*P*
1	4.00	0.288	12	0.27	1.43	0.20
2	1.33	0.096	3	0.27	1.42	0.21
3	1.07	0.077	0	0.27	1.61	0.20
4	0.80	0.058	0	0.27	1.41	0.18
5	0.67	0.048	0	0.27	1.21	0.15
6	0.53	0.038	0	0	1.01	0.13
7	0.40	0.029	0	0	0.81	0.10
8	0.27	0.019	0	0	0.60	0.08
9	0.13	0.010	0	0	0.40	0.05
10	0.07	0.005	0	0	0.20	0.03

Partial harvesting in mineral soil forests is assumed not to increase N and P exports, and fertilization with P in mineral soil forests does not increase P exports (Finér et al. 2010). Export coefficients for those operations are therefore 0 kg ha^−1^ year^−1^. The N export coefficient for fertilization of drained peatland forests is also 0 kg ha^−1^ year^−1^ because drained peatlands are not fertilized with N (Finér et al. 2010)

^a^Calculated as the product of DNM-induced suspended solid exports in Finér et al. (2010) and N and P concentrations in suspended solids in Nieminen et al. (2017a)

^b^Finér et al. (2010)

^c^Nieminen et al. (2023)

The data used for calculating these N and P export coefficients (Table 2) is sourced from 37 catchments for DNM (Joensuu et al. 1999), 10 catchments for clearcutting of mineral soil forests (Finér et al. 2010, Nieminen et al. 2023), 15 catchments for fertilizing drained peatland forests (Piirainen et al. 2013), and one catchment for fertilization of mineral soil forests (Finér et al. 2010). All catchments are located in Finland. When estimating the effect of fertilization on nutrient exports, it is further assumed that mineral soil forests are fertilized with ammonium nitrate and drained peatland forests with wood ash, as these are currently the only commercially available forest fertilizers in Finland.

The effect of harvesting on annual N and P exports (*N*_*harvest*_ and *P*_*harvest*_, kg ha^−1^ year^−1^) from drained peatland forests was calculated as the relationships between harvested stem volume (*V*_*cut*_, m^3^ ha^−1^) and harvest-induced N and P exports as in Nieminen et al. (2023).

2 $${N}_{{harvest}}=\frac{10}{1+{e}^{-\left(-3.3375\,+\,0.0164{V}_{{cut}}\right)}}$$

3 $${P}_{{harvest}}=\frac{1.3}{1+{e}^{-\left(-5.1772\,+\,0.0209{V}_{{cut}}\right)}}$$

Thus, it was assumed that the effect of harvesting on N and P exports from drained peatland forests is more closely related to the stem volume of the removed growing stock than the harvesting method (clear-cut, thinning, CCF harvest). The data by Nieminen et al. (2023) consist of 16 harvested peatland catchments, of which 15 are in Finland and one is in Ireland. As in Nieminen et al. (2023), harvesting was assumed to increase N and P exports for 4 years from low-productive sites (sub-xeric or poorer), and for 6 years from fertile peatland sites (mesic or better).

#### Water Quality Protection Practices

The effect of water protection practices on the reduction of forestry-induced nutrient loads in our calculations is explained below, and the original data points used for calculating the reduction efficiencies are given in the appendix (Appendix Figs. 8–10).

**Fig. 1:**
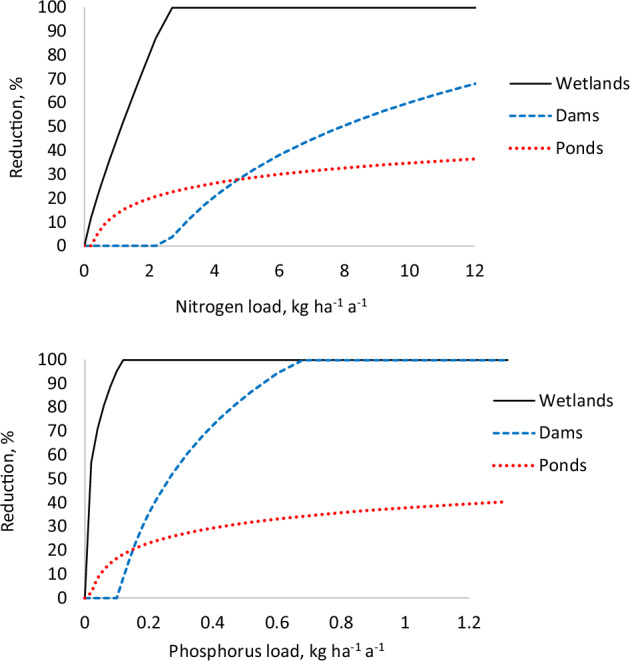
Reduction of N and P exports by wetland buffers, peak runoff control dams, and sedimentation ponds as percentages of their loads above them

The effect of sedimentation ponds on the retention of DNM-induced P and N exports (Water protection practice 1, Table 3) was calculated using the data by Nieminen et al. (2018b) for suspended solid exports above and below 37 ponds. We assumed that sedimentation ponds only decrease the export of particulate N and P. The percentage retention of these nutrients was estimated by multiplying the suspended solid exports measured upstream and downstream of 37 sedimentation ponds by the average concentrations of N and P in suspended solids reported by Nieminen et al. (2017a). We further assumed that the reduction does not get negative values, that is, we assumed zero reduction instead of negative reduction with low N and P loads above the ponds (Figs. 1, 8). Our calculations indicated that sedimentation ponds may, at their peak, retain about 40% of the N and P loads above the ponds.

**Table 3:** Scenarios for water protection practices in the Kiiminkijoki catchment area

Scenario No. for water protection practice	Water protection practice
1	Sedimentation pond^a^
2	Peak runoff control dam^a^
3	Natural wetland buffer at each drained peatland
4	Riparian buffer zone at each mineral soil forest

^a^Only in conjunction with ditch network maintenance (DNM)

The effect of peak runoff control dams on the decrease in DNM-induced N and P exports (Water protection practice 2, Table 3) was calculated using the data by Amatya et al. (2003) and Marttila and Kløve (2010). As for sedimentation ponds, we calculated the decrease in N and P exports by peak runoff control dams as the percentage of N and P exports above them. The study by Marttila and Kløve (2010) did not involve N and P exports above their dam. Therefore, we assumed that the N and P exports above the dam in the Virkonrinne catchment were similar to the N and P exports in the Virkonsuo reference catchment. As for sedimentation ponds, we assumed that the reduction is never negative, but it can be zero with low N and P loads above the dams (Figs. 1, 9).

The effect of wetland buffer areas on forestry-induced N and P exports from drained peatland forests (Water protection practice 3, Table 3) was calculated using the data by Sallantaus (2023). We subtracted “not-by-forestry-induced” background exports from the exports above and below wetland buffers and then calculated the retention of forestry-induced exports as the percentage of the exports above the wetland buffer (Figs. 1, 10). Background export for N was assumed to be 1.3 kg ha^−1^ yr^−1^, and that for P, 0.025 kg ha^−1^ yr^−1^.

The effect of buffer zones on N and P exports from mineral soil forests (Water protection practice 4, Table 3) was estimated using the relationship by Miettinen et al. (2020) for nutrient retention (% of loading) and percentual coverage of buffer zones in the catchment. We estimated, based on Räty et al. (2022), that traditional 5–15 m wide buffer zones cover, on average, about 3% of the catchment in forested areas, and therefore retain about 12% of forestry-induced nutrient loading (Miettinen et al. 2020). Miettinen et al. (2020) indicated that nutrient retention is not particularly sensitive to the coverage (or width) of the buffer zones, as doubling the area of a buffer zone increases nutrient retention only by about 5%.

### Simulation of Scenarios

The future development of each stand of the Kiiminkijoki catchment area was simulated for 50 years, divided into five 10-year periods, using the Monsu simulation and planning software (Pukkala 2011). Metsähallitus and Forestry Center datasets, which cover about 93% of the land area in the Kiiminkijoki catchment area, were utilized as data sources for the initial status of the forests.

Cuttings and other treatments were simulated in the middle of each 10-year period. The individual-tree models of Pukkala et al. (2021) were used to simulate stand dynamics, i.e., diameter increment of trees, survival, and ingrowth. Tree heights were calculated using static individual tree height models. Stem volumes of trees were calculated with the taper models of Laasasenaho (1982).

In the RF management scenario (Table 1), a final felling was simulated when the mean tree diameter exceeded the recommended minimum diameter for final felling (Äijälä et al. 2014). The final felling was clear-cutting except in pine-dominated stands on xeric sites where natural regeneration via seed trees was used. Site preparation and artificial regeneration were simulated immediately after clear-felling.

Seeding for pine was assumed on sub-xeric sites, and planting on more fertile sites. On mesic sites, the probabilities of planting pine, spruce or silver birch were 0.3, 0.6 and 0.1, respectively. On more fertile sites, the corresponding probabilities were 0 for pine, 0.9 for spruce and 0.1 for birch. Natural regeneration of several broadleaf species, such as downy birch, silver birch, rowan and aspen, was assumed, in addition to the planted trees. The amount and species composition of natural regeneration depended on site fertility. Tending treatments of the newly regenerated stands were simulated during the second 10-year period.

Commercial thinning treatments were simulated when the stand basal area exceeded the “thinning limit” of the silvicultural recommendation (Äijälä et al. 2014) and the stand was not mature for final felling. The stand basal area was decreased to the recommended post-thinning level. The thinning was simulated as thinning from below. Half of the removed basal area was removed by using the same thinning percentage in all diameter classes. The other half was obtained by removing the smallest trees until the required basal area reduction was reached.

The second scenario (ERP) for mineral soil used extended rotation lengths and thinning from above (Table 1). Extended rotations were simulated by increasing the mean tree diameter required for final felling by 10%. Thinning from above was simulated so that half of the removed basal area was taken using the same thinning intensity in all diameter classes (as in thinning from below). The other half of the basal area reduction was obtained by removing the largest trees of the stand. Since thinning from above decreases the mean diameter, it postpones the final felling even when the threshold diameter for the final felling is not altered. The combined effect of increasing the final felling diameter and switching from thinning from below to thinning from above may therefore be substantial.

Simulation of CCF cuttings used optimization-based guidelines, where the threshold basal area for cutting is determined as a function of mean tree diameter, site fertility, temperature sum, and discount rate (Pukkala 2022). The guidelines also indicate the thinning intensity for different diameter classes. Since the thinning intensity increases toward larger diameter classes, CCF cutting corresponds to thinning from above. CCF scenarios were simulated for mineral soils and peatland forests using the same guidelines in both cases. The guidelines were applied with a 3% discount rate.

Nitrogen fertilization was simulated in even-aged management on mineral soils in pine-dominated xeric sites and spruce-dominated mesic sites if the mean tree diameter was 23–33 cm and the stand basal area was 15–40 m^2^ ha^−1^. The time since the previous fertilization treatment had to be more than 10 years. The outcome of these parameters was that there was one fertilization treatment during the rotation, and fertilization was simulated when the stand approached maturity for final felling.

Peatland fertilization was simulated in RF and CFF when the mean tree diameter was 5–30 cm and the stand basal area was 10–40 m^2^ ha^−1^. The time since the previous fertilization had to be more than 50 years. This means that a peatland stand could be fertilized once during the 50-year simulation period.

Ditch network maintenance was simulated in peatland forests after final felling, and if the mean tree diameter was 5–30 cm, basal area 10–40 m^2^ ha^−1^ and at least 30 years had elapsed since previous ditch network maintenance. However, ditch maintenance was not simulated if the growing stock volume exceeded 150 m^3^ ha^−1^. This is because evapotranspiration of such mature stands is assumed to keep the water level sufficiently low for undisturbed tree growth, even when the condition of the ditch network is poor (Sarkkola et al. 2010).

Nitrogen fertilization and ditch network maintenance improved tree growth. The response functions were the same as described in Heinonen et al. (2018). Peatland fertilization was used to mitigate nutrient shortages and maintain the normal “without-nutrient-shortages” growth level.

All planted trees were assumed to grow 10% faster than naturally regenerated trees and trees of artificial seeding origin were assumed to grow 5% faster. These “tree breeding benefits” were implemented by multiplying the prediction of the diameter increment model by 1.1 (planted tree) or 1.05 (tree from artificial seeding).

## Results

**Fig. 2:**
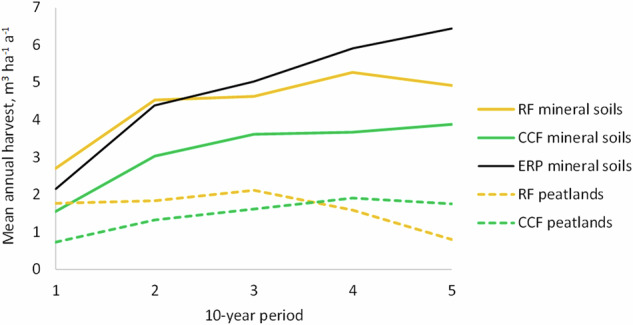
Simulated mean annual harvest volumes in mineral soil and peatland forests of the Kiiminkijoki catchment over 50 years according to different forest management scenarios. RF rotation forestry, CCF continuous cover forestry, ERP extended rotation period

The simulated harvest scenarios indicated smaller harvest volumes for ERP than RF management in mineral soil forests during the next 20 years, but somewhat larger after that (Fig. 2). CCF was less intensive in terms of harvest volumes than the RF and ERP scenarios during the entire simulated 50-year time span. For drained peatland forests, simulated harvest volumes were lower in the CCF scenario than in the RF scenario during the next 30 years, but higher during the fourth and fifth 10-year period.

**Fig. 3:**
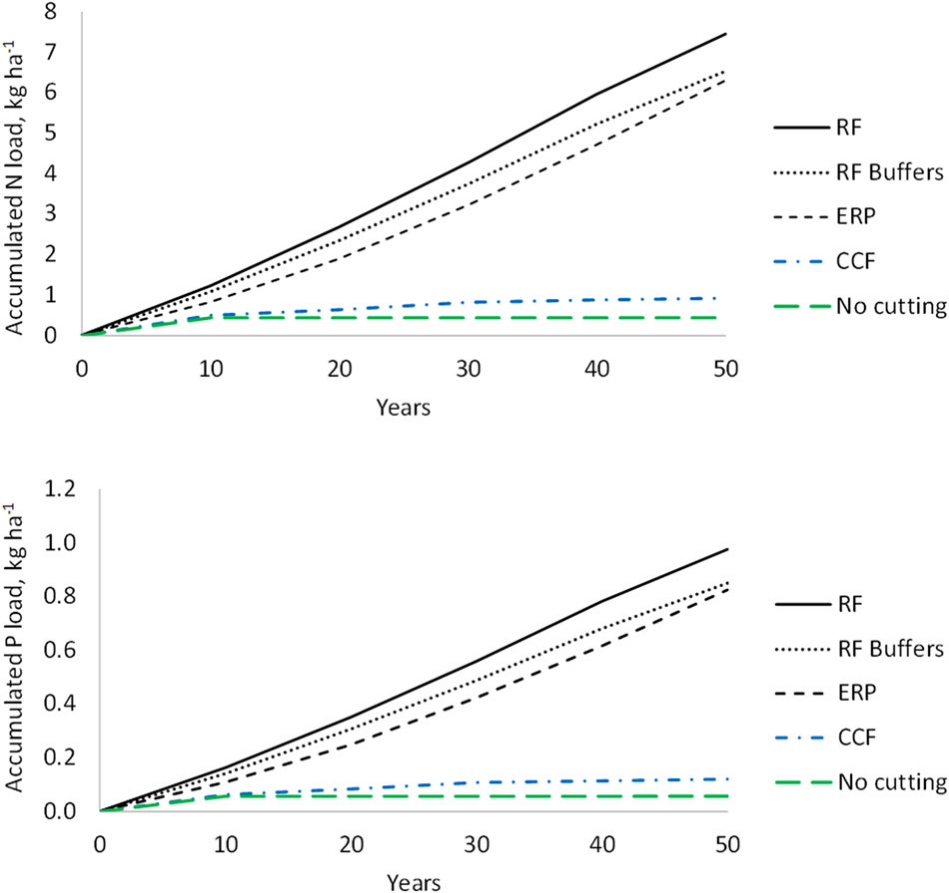
Simulated accumulated N and P exports from mineral soil forests of the Kiiminkijoki catchment area over 50 years with different scenarios for forest management and water protection measures. RF rotation forestry, RF Buffers rotation forestry and riparian buffer zone at each mineral soil forest, ERP extended rotation period, CCF continuous cover forestry, No cutting = no forest operations

In terms of N and P exports to water courses, two distinctly different patterns could be observed in mineral soil forests (Fig. 3). The RF scenario, with and without buffer zones, and the ERP scenario resulted in significantly higher exports than the CCF and no forestry scenarios. Moreover, while N and P exports from the first ones indicated no considerable variation during the simulated 50-year time span, the latter scenarios indicated any exports only during the first 10-year period. These exports for CCF and no forestry scenario are due to “residual exports” of the clear-cuts that had been done before the first 10-year period.

**Fig. 4:**
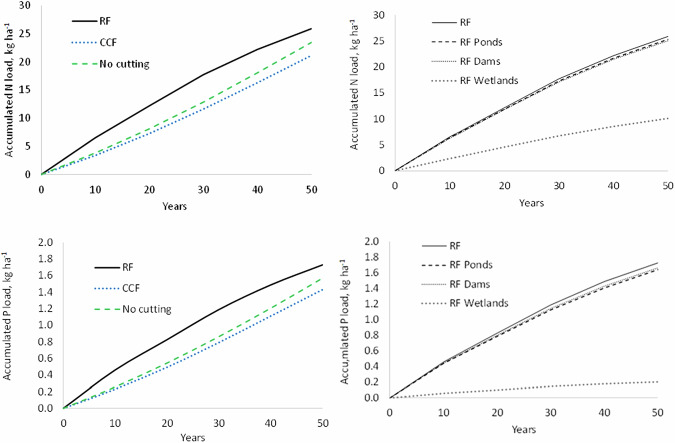
Simulated accumulated N and P exports from drained peatland forests of the Kiiminkijoki catchment area over 50 years with different scenarios for forest management and water protection measures. RF rotation forestry, CCF continuous cover forestry, No cutting = no forestry operations, RF Ponds = rotation forestry and sedimentation pond in each drained peatland forest, RF Dams = rotation forestry and peak runoff control dam in each drained peatland forest, RF Wetlands = rotation forestry and wetland buffer in each drained peatland forest

In drained peatland forests, the RF scenario increased N and P exports more than the CCF and no forestry scenarios, particularly during the first 20–30 years (Fig. 4). N and P exports in the RF scenario in drained peatland forests were not much influenced by either sedimentation ponds or peak runoff control dams, but wetland buffers decreased the exports to a significantly lower level.

**Fig. 5:**
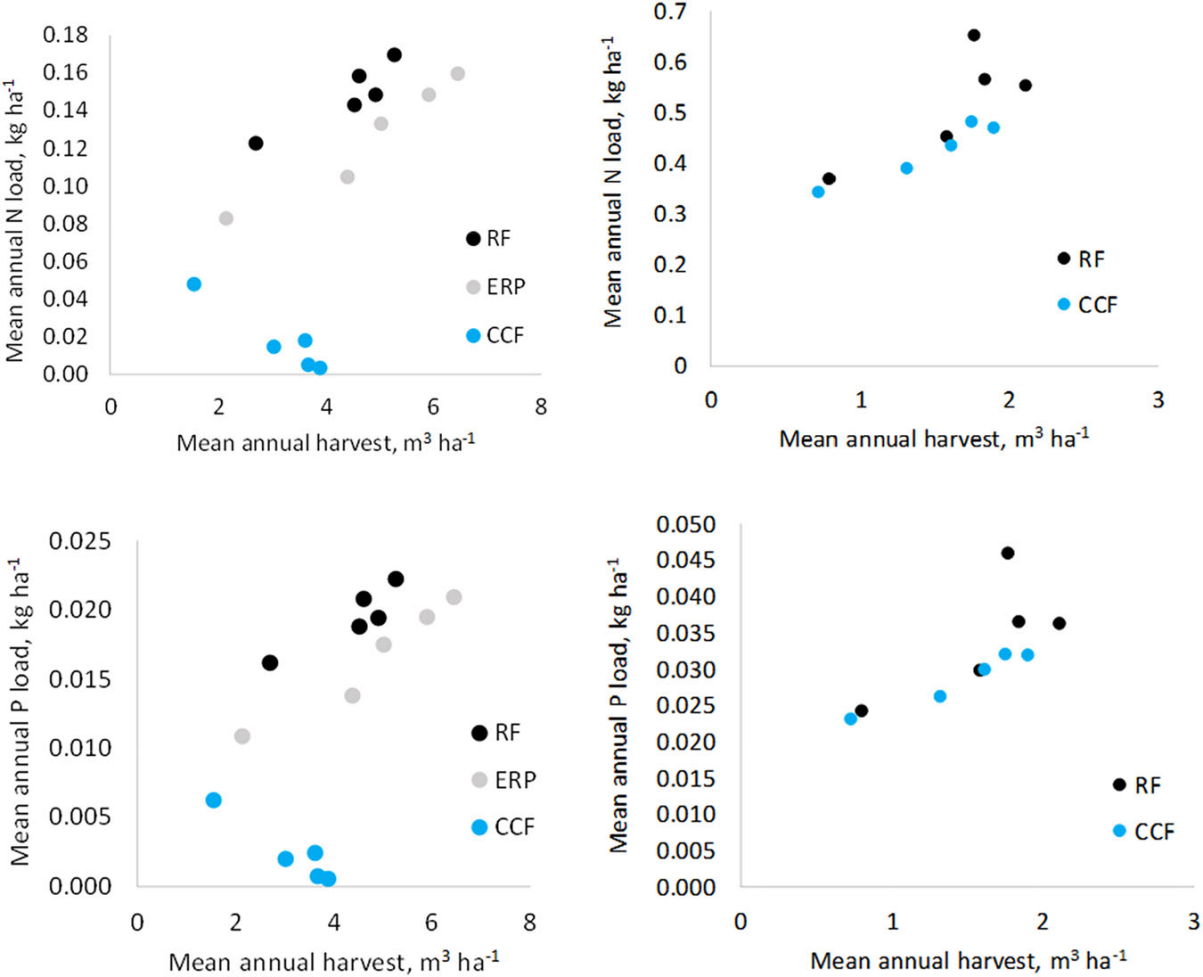
Simulated relationships between annual harvest volumes and mean annual N and P exports in mineral soils (left) and peatlands (right) of the Kiiminkijoki catchment area with different scenarios for forest management. RF rotation forestry, ERP extended rotation period, CCF continuous cover forestry

**Fig. 8:**
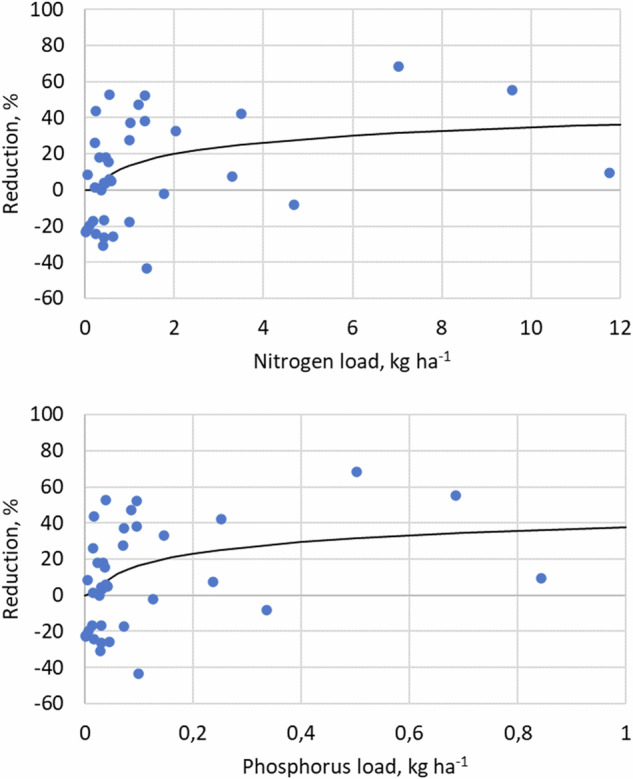
Reduction of N and P loads by sedimentation ponds as a function of their loads above ponds. Percentual reduction of N and P loads by sedimentation ponds were calculated by multiplying suspended solid exports above and below 37 ponds in the data by Nieminen et al. (2018b) with the average concentrations by Nieminen et al. (2017a) for N and P in suspended solids

**Fig. 9:**
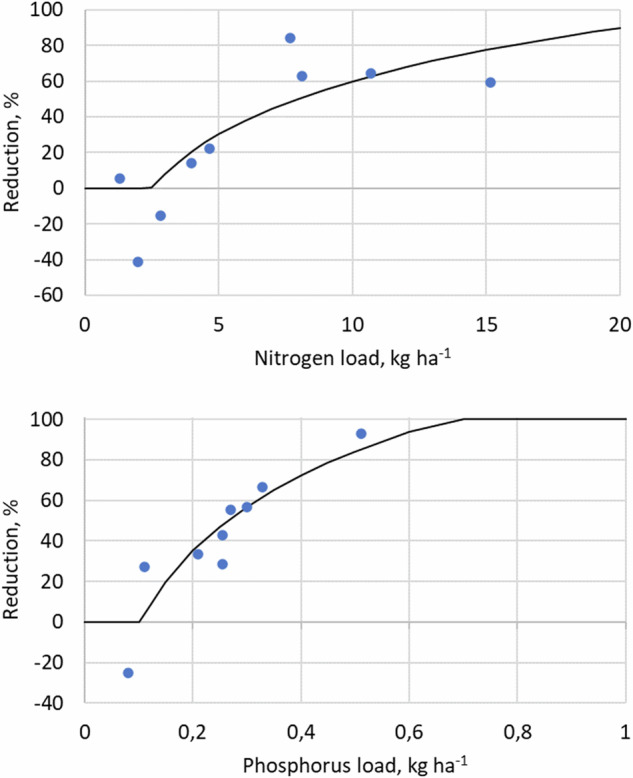
Reduction of N and P loads by peak runoff control dams as a function of their loads above dams. Calculated using the data by Amatya et al. (2003) and Marttila and Kløve (2010). For further explanation of the calculation of the efficiency of peak runoff control dams, see the text

Simulated N and P exports in mineral soil forests in RF and ERP scenarios were positively related to the rate of mean annual harvest of 10 years, but the relationship between N and P exports and harvest volumes was less clear in the CCF scenario (Fig. 5). The N and P exports for CCF with low harvest volumes are again due to “residual” exports of the clear-cuts that had been done before the first 10-year period. The simulation data for drained peatland forests indicated a positive relationship between harvest volume and nutrient exports, both in the RF and CCF scenarios.

## Discussion

A general problem in improving water quality management in forested areas is that there are multiple alternative options to decrease nutrient exports, such as implementing CCF or increasing the rotation length in RF. There are also several water quality management methods, such as sedimentation ponds, runoff control dams, riparian buffer zones, or wetland buffers. Owing to these multiple options to manage water quality, there is no general agreement on how forests should be managed, and which water protection practices should be implemented to steer forestry in a more environmentally friendly direction. We executed a simulation study to assess the effects of alternative forest management options and water quality protection practices on nutrient exports concurrently from the same catchment. Even though such simulations involve uncertainties (see below), our approach of introducing different management options and protection practices at the same space and time paves the way for a more holistic understanding of achieving good water quality in forested catchments.

It is to be noted that our approach is not restricted to boreal forest ecosystems, but it can be extended to broader geographical contexts to improve water quality management in forested catchments, provided that sufficient data is available on the mechanisms affecting nutrient loads. Nevertheless, the need for improved water quality management is particularly urgent in catchments dominated by drained peatland forests, where our simulation model is currently most applicable.

Simulation is the only feasible way to forecast the effects of different forest management options and water protection practices on nutrient exports, especially when aiming to facilitate water quality management in a large catchment. This is particularly true when assessing the combination of various management options and/or water protection practices, as the number of treatments may become impractically large for an empirical study (Haahti et al. 2018). Moreover, as regards the uncertainty of our results, our aim has not been to produce exact N and P export estimates but to identify which forest management and water protection practices should be prioritized in future research and operational forestry from a water quality management viewpoint.

Our simulation study indicated that nutrient exports from a large boreal forest catchment are most efficiently reduced by not managing mineral soil forests or managing them with CCF instead of RF, as well as using wetland buffer areas as a water protection practice in drained peatland forests. In contrast, traditional buffer zones, sedimentation ponds, and peak runoff control dams were inefficient in decreasing nutrient exports. Our study similarly indicated that nutrient exports may increase with increasing harvest intensity, except when mineral soil forests are managed by CCF.

### Forest Management Scenarios

Our simulation indicated that the differences in nutrient exports from drained peatland forests between different forest management options vary considerably over time. For example, while RF increased nitrogen exports by about 0.55 kg ha^−1^ year^−1^ and CCF by about 0.35 kg ha^−1^ year^−1^ during the first 10-year period, the fifth 10-year period indicated an increase by about 0.35 kg ha^−1^ year^−1^ for RF and 0.50 kg ha^−1^ year^−1^ for CCF. This contrasting behavior over time may be because nutrient exports from drained peatland forests are governed by harvesting and the long-term legacy effect of drainage. When peatland forests are mature and are intensively harvested, as for the RF scenario during the next three decades (Fig. 2), nutrient exports are high due to harvest-induced exports but also because of the high evapotranspiration capacity of the remaining mature stands. This high evapotranspiration results in low WTLs (Sarkkola et al. 2010) and faster-than-average peat mineralization (Ojanen and Minkkinen 2019), which increases nutrient mobilization and their release to ditch outflow waters (Laurén et al. 2021; Nieminen et al. 2022). After the drained sites in the RF scenario have been clear-cut and harvest-induced exports from them have returned to pre-harvest levels, nutrient exports may be even lower than for the CCF and no forestry scenarios due to the small evapotranspiration capacity of young seedling and sapling stands. This results in high WTLs and slow peat mineralization and nutrient mobilization. Nutrient exports in the RF scenario would increase again (after 50 years) when the forests mature and their evapotranspiration increases.

The environmental usefulness of CCF compared to RF in drained peatland forests depends very much on how often and how intensively CCF forests are harvested, as was shown for greenhouse gas emissions by Shanin et al. (2021). In our scenario calculations, CCF forests are harvested less intensively than forests in the RF scenario (logging volume 0.16 m^3^ ha^−1^ year^−1^ lower), which may decrease their harvest-induced exports but increase the exports caused by low WTLs and mineralization of deep peat layers.

There is no direct empirical evidence that nutrient exports from drained peatland forests are controlled by their WTLs and nutrient mineralization similarly as in our calculations. Even though there is no empirical data, many studies indicate that WTL and nutrient mineralization may affect nutrient exports from drained peatlands. The studies by Nieminen et al. (2017b, 2018c, 2022) have shown that nutrient concentrations discharging from drained peatlands are high from mature stands where WTLs are low (Sarkkola et al. 2010) and where peat mineralization is therefore faster than for sites with high WTL (Ojanen and Minkkinen 2019). The study by Nieminen et al. (2022) also indicated that there is a positive correlation between peat bulk density and nutrient concentrations discharging from drained peatland forests. Peat bulk density in a peatland may only be high if it has been properly drained, that is, if its WTL has been lowered and if aerobic peat mineralization has increased. Nevertheless, future studies should concurrently monitor WTLs, peat mineralization and nutrient exports in a variety of drained peatland forests to confirm the results of our calculations.

As for the empirical coefficients and equations utilized in our study, relatively large datasets were used for their estimation, except for the export coefficient for fertilization of mineral soil forests, which was based on only one catchment. For example, the prediction of WTL in drained peatland forests utilized data from 460 sample plots (Eq. 1; Sarkkola et al. 2010). The export coefficient for harvesting of mineral soil forests is derived from 10 catchment experiments, while the fertilization export data for peatland forests comes from 15 catchments (Table 2). The data used for calculating export coefficients and models are from Finland, with the exception that one site in Ireland contributed to the relationship between harvested stem volume and harvest-induced P exports from drained peatland forest (Rodgers et al. 2010; Nieminen et al. 2023). Therefore, the data should predict relatively well the effects of different forestry operations on nutrient exports in the study area, although it is obvious that more data would reduce the uncertainty associated with the nutrient export estimates, particularly the N exports caused by the fertilization of mineral soil forests.

According to our simulations, extending the rotation length (ERP scenario) decreased nutrient exports from mineral soil forests only slightly compared with the RF scenario. This is because harvesting is the main source of nutrient exports from mineral soil forests and harvest volumes in the long term do not differ much between RF and ERP (Fig. 2).

CCF in mineral soil forest was almost as efficient a management option in decreasing nutrient exports as avoiding all forestry operations. This is mostly because of the assumption of this and many earlier studies (Finér et al. 2010; Nieminen et al. 2023) that less intensive harvestings than clear-cuts do not increase nutrient exports from mineral soil forests. There is no empirical data to support that assumption, but Palviainen et al. (2014) indicated that even clear-cuts may not always increase nutrient exports from mineral soils if they leave a large unharvested area within the catchment and remove significantly less wood than large-scale clear-cuts. However, as long as there are no studies on nutrient exports from mineral soil forests treated with harvestings other than clear-cuts, the performance of CCF compared with RF management will remain incompletely understood.

In the interpretation of the results, it should also be noted that forest harvesting in Monsu simulations are based on silviculture recommendations and not on the demand for wood by the forest industry and energy sectors. Our simulations may therefore have sometimes harvested more and sometimes less timber than the current harvest rate by the Finnish forest and energy sectors. As shown by the relationship between harvest volumes and nutrient exports, nutrient exports were positively related to harvest volumes, except for CCF in mineral soil forests (Fig. 5).

### Water Quality Management Scenarios

Our simulation study indicated that riparian buffer zones, sedimentation ponds, and peak runoff control dams may have little effect on nutrient exports from the Kiiminkijoki catchment area. This is because they are either relatively inefficient in reducing nutrient exports (sedimentation ponds, riparian buffer areas) or because they only decrease DNM-induced particulate nutrient exports (sedimentation ponds, peak runoff control dams) and DNM operations constitute annually less than 1% of the Kiiminkijoki area. In the large catchments, the water protection practices targeted to be used only in conjunction with a specific forestry operation, such as DNM, which is executed in a small area, may therefore not be particularly efficient. Poor performance of the water protection practices, such as sedimentation ponds and peak runoff control dams, is also because they are not efficient in decreasing nutrient exports when DNM-induced nutrient loadings are low. Such high loadings, when sedimentation ponds and peak runoff control dams are efficient, generally occur only one-to-two years after DNM (Joensuu et al. 1999, 2002; Nieminen et al. 2010).

**Fig. 10:**
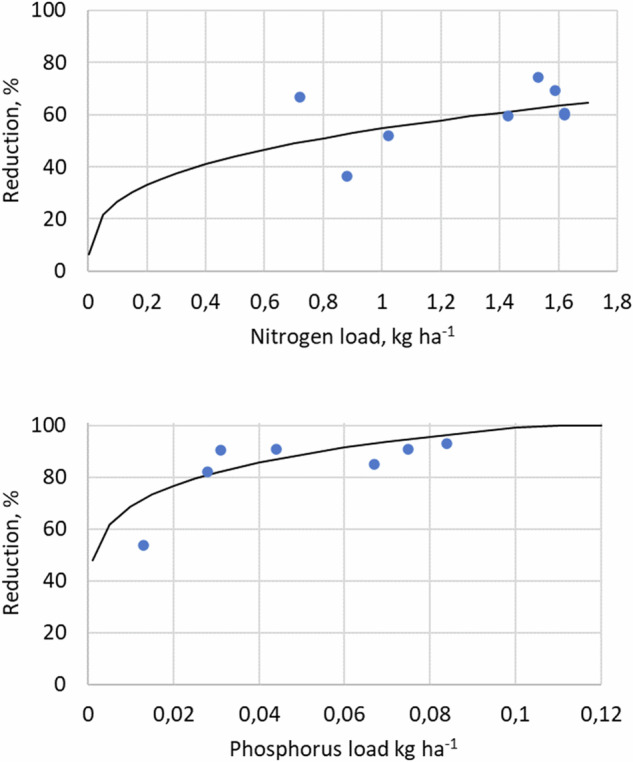
Reduction of N and P loads by wetland buffers as a function of their loads above buffers. Modified from the data by Sallantaus (2023) by subtracting background loads from the original N and P loads. For further explanation of the calculation of the efficiency of wetland buffers, see the text

Instead, wetland buffers in drained peatland forests were a highly efficient means of decreasing nutrient exports. The greatest uncertainty in the behavior of wetlands in nutrient reduction in our calculations relates to N reduction with low N loads above wetlands. This is because the data used for estimating the efficiency of wetland buffers in reducing N exports lacked loads lower than 0.6 kg ha^−1^. It is thus unclear whether the efficiency of wetland buffers under low N loads is as indicated by Fig. 10.

Our study also assumed that the waters from each drained peatland forest are conveyed to receiving water bodies over a natural wetland buffer. As wetlands have often been drained for forestry, agriculture, or peat mining purposes, there may be no suitable natural wetlands to be used as buffer areas. A common practice is therefore to restore sections of drained peatlands by filling in or blocking their drainage ditches. This blocking or filling in the ditches in the area planned to be used as a wetland buffer creates a problem that WTL does not rise only in the buffer area itself, but also in its upstream catchment area, potentially reducing tree growth and vitality there. Thus, even though wetland buffers may be a highly efficient means of decreasing diffuse pollution in forested catchments, their use in flat areas is complicated due to potential negative effects on their upstream productive forest.

Constructing a wetland buffer by restoration also creates a problem that restored buffers initially increase rather than decrease nutrient exports (Sallantaus et al. 1998; Nieminen et al. 2020b). The duration of this initial restoration-induced excess export phase is still poorly understood. Another major limitation in the use of buffer areas arises from the need to conserve endangered wetland site types. The use of these sites as buffer areas may induce unwanted changes in the plant species composition (Hynninen et al. 2011). Mires with high conservation values should therefore be left aside from buffer use and water quality protection. On the other hand, the hydrology of many natural or nearly natural mires may be affected by drainage of surrounding areas (Sallinen et al. 2019), so directing waters to such mires may contribute positively to their hydrology and vegetation (Isoaho et al. 2023).

Even though there are limitations in the use of wetland buffers, it should be noted that they are such an effective water protection practice that they should be favored over any other method, wherever and whenever possible. In our simulations, they decreased forestry-induced exports only from drained peatland forests, but wherever they are used, they, of course, also retain the nutrients from the surrounding upstream mineral soil forests of the same catchment. In the use of wetland buffers as a water protection practice, it should also be noted that very small buffers (<1–2% of the catchment area) are significantly less efficient in reducing nutrient exports than larger wetland buffers (Väänänen et al. 2008; Vikman et al. 2010). Our results on the efficiency of wetland buffers were based on Sallantaus’ (2023) study with relatively large natural wetlands (13–37% of catchment area), which should be considered in the interpretation of the results. How large wetland buffers are needed for the efficient reduction of forestry-derived nutrient exports still needs future research, as most of the data collected so far are from relatively small buffers (Väänänen et al. 2008; Vikman et al. 2010).

### Future Research

Besides increasing information on the effects of wetland buffers on nutrient reduction, future research is needed concerning other factors related to forest management and water quality protection practices. Validation of the results of SUSI simulations on the relationship between WTL and nutrient exports requires concurrent data on WTLs, peat mineralization, and nutrient exports from drained peatland forests under varying environmental conditions. Future research should also monitor the nutrient exports caused by ditch-mounding, which is becoming a more and more popular drainage and soil preparation option in Finnish forests, but which so far has received little attention as a potential source of nutrients in water bodies. Ditch-moundings, especially in fine-textured soils, may considerably increase the exports of suspended solids and adhered nutrients (Nieminen 2003). Future research should also increase the understanding of partial harvests on nutrient exports from mineral soil forests. It has so far been assumed that they do not increase nutrient exports, but there are no empirical studies to confirm that assumption.

As for the water protection practices, information is also needed on the effects of restored wetland buffers on nutrient exports. A key question is how long it takes for a restored wetland buffer to turn from an initial source of nutrients to a sink. This does not necessarily take a very long time as high nutrient inflows from the upstream catchment may be retained already when restoration still increases nutrient mobilization and leaching from rewetted peat. By combining the data from Sallantaus et al. (1998) and Nieminen et al. (2020b), we estimated (data not shown) that significant nutrient exports from the restoration operations of wetland buffers may last only 5–6 years. However, whether they then act as equally efficient nutrient sinks as natural wetlands still requires future research.

## Conclusions

We used empirical and process-model-based nutrient export coefficients and models and forestry simulations to forecast the impact of forest management and water protection practices on nitrogen and phosphorus exports from forests to waters in the Kiiminkijoki catchment area, central Finland.

The main conclusions based on simulations are:

- The choice between forest management systems (even-aged forestry, extended rotation length, continuous cover forestry, no forestry) may have a greater impact on nutrient exports from mineral soil forests, as compared to drained peatland forests
- Increasing tree harvests increase nutrient exports, except for CCF in mineral soils
- Of the water protection practices, sedimentation ponds, peak runoff control dams and riparian buffer zones may have little effect on nutrient exports from a large catchment area, either because they are relatively inefficient in reducing nutrient exports (sedimentation ponds, riparian buffer zones) or because they only decrease particulate nutrient exports from DNM-treated drained peatland forests (sedimentation ponds, peak runoff control dams)
- Wetland buffers in drained peatland forests may be a highly efficient means of decreasing nutrient exports
- Integration of forest simulation models and empirical nutrient export coefficients and models may provide a powerful tool to facilitate the planning and implementation of water quality management solutions in a large catchment

## Figures and Tables

**Fig. 6:**
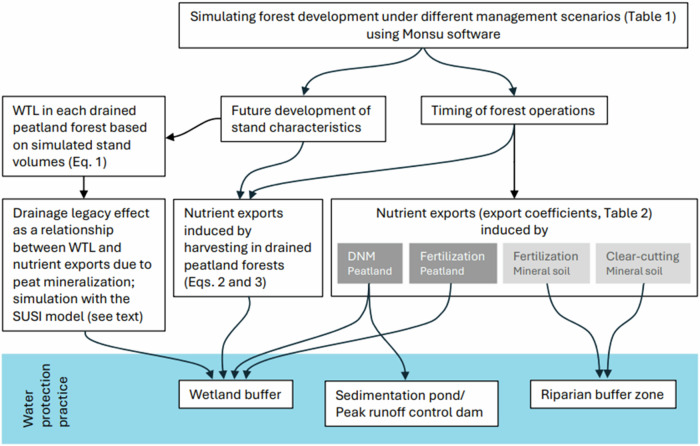
Flow chart for the calculation procedure for estimating nutrient exports for the Kiiminkijoki catchment area. WTL = water table level in soil in each drained peatland forest, DNM ditch network maintenance. Water protection practice in DNM-treated areas was either sedimentation pond, peak runoff control dam, or wetland buffer. Simulations for the other forest operations involved only one water protection practice

**Fig. 7:**
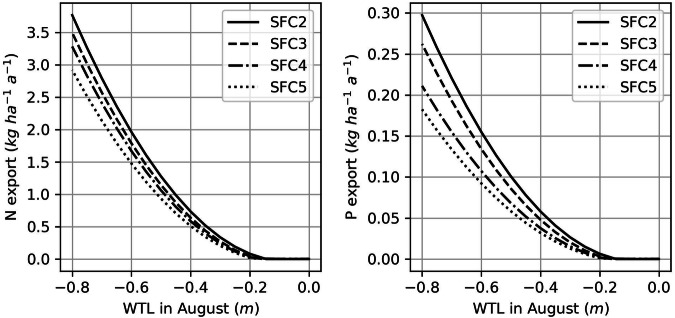
The relationships between WTLs during late summer (August) and the N and P exports caused by the long-term legacy effect of past drainage operations as simulated by the SUSI model (Laurén et al. 2021). Site fertility classes: SFC2 = Eutrophic and herb-rich sites, SFC3 = Vaccinium myrtillus sites, SFC4 = Vaccinium vitis-idaea sites, SFC5 = Dwarf shrub and poorer site types

## Data Availability

No datasets were generated or analyzed during the current study.

## Appendix

Figs. 6–10
